# Synergistic Killing and Re-Sensitization of *Pseudomonas aeruginosa* to Antibiotics by Phage-Antibiotic Combination Treatment

**DOI:** 10.3390/ph14030184

**Published:** 2021-02-25

**Authors:** Emily Engeman, Helen R. Freyberger, Brendan W. Corey, Amanda M. Ward, Yunxiu He, Mikeljon P. Nikolich, Andrey A. Filippov, Stuart D. Tyner, Anna C. Jacobs

**Affiliations:** 1 Center for Infectious Diseases Research, Wound Infections Department, Bacterial Diseases Branch, Walter Reed Army Institute of Research, Silver Spring, MD 20910, USA; emilytengeman@gmail.com (E.E.); hrfreyberger@gmail.com (H.R.F.); amanda_ward45@yahoo.com (A.M.W.); yunxiu.he.ctr@mail.mil (Y.H.); mikeljon.p.nikolich.civ@mail.mil (M.P.N.); stuart.d.tyner.mil@mail.mil (S.D.T.); jacobs.anna.c@gmail.com (A.C.J.); 2 Oak Ridge Institute for Science and Education (ORISE), Oak Ridge, TN 37831, USA; 3 ICON plc, Ellicott City, MD 21043, USA; 4 Multidrug-Resistant Organism Repository and Surveillance Network, Bacterial Diseases Branch, Center for Infectious Diseases Research, Walter Reed Army Institute of Research, Silver Spring, MD 20910, USA; brendan.w.corey.ctr@mail.mil

**Keywords:** *Pseudomonas aeruginosa*, phage therapy, phage–antibiotic synergy, antimicrobial resistance, re-sensitization

## Abstract

Multidrug-resistant (MDR) *Pseudomonas aeruginosa* infections pose a serious health threat. Bacteriophage–antibiotic combination therapy is a promising candidate for combating these infections. A 5-phage *P. aeruginosa* cocktail, PAM2H, was tested in combination with antibiotics (ceftazidime, ciprofloxacin, gentamicin, meropenem) to determine if PAM2H enhances antibiotic activity. Combination treatment in vitro resulted in a significant increase in susceptibility of MDR strains to antibiotics. Treatment with ceftazidime (CAZ), meropenem, gentamicin, or ciprofloxacin in the presence of the phage increased the number of *P. aeruginosa* strains susceptible to these antibiotics by 63%, 56%, 31%, and 81%, respectively. Additionally, in a mouse dorsal wound model, seven of eight mice treated with a combination of CAZ and PAM2H for three days had no detectable bacteria remaining in their wounds on day 4, while all mice treated with CAZ or PAM2H alone had ~10^7^ colony forming units (CFU) remaining in their wounds. *P. aeruginosa* recovered from mouse wounds post-treatment showed decreased virulence in a wax worm model, and DNA sequencing indicated that the combination treatment prevented mutations in genes encoding known phage receptors. Treatment with PAM2H in combination with antibiotics resulted in the re-sensitization of *P. aeruginosa* to antibiotics in vitro and a synergistic reduction in bacterial burden in vivo.

## 1. Introduction

The discovery of penicillin as an antimicrobial in 1928 by Alexander Fleming launched the world into a new era of treating infectious diseases [1]. The antibiotic “golden era” was hallmarked by the development of multiple classes of antibiotics that were effective for decades in controlling infections and reducing morbidity [2]. However, as widespread use of antibiotics increased globally, bacterial pathogens accumulated mechanisms to evade antimicrobial treatments. The emergence of multidrug-resistant (MDR) bacteria coupled with the waning of the antibiotic development pipeline has moved society back into an era where bacterial infections pose a serious threat to human health [3,4].

In 2004, the Infectious Diseases Society of America (IDSA) published the list of ESKAPE pathogens (*Enterococcus faecium, Staphylococcus aureus, Klebsiella pneumoniae, Acinetobacter baumannii, Pseudomonas aeruginosa*, and *Enterobacter* species) that have become increasingly resistant to antibiotics and cause the bulk of nosocomial infections in hospital settings [5,6]. As a result, the ESKAPE pathogens have become a top target for novel antimicrobial therapies. Among these species is *P. aeruginosa*, a gram-negative, opportunistic bacterium that can cause complex recurrent infections, particularly in immunocompromised groups [7].

*P. aeruginosa* has acquired multiple mechanisms to evade treatment by current antibiotics including biofilm formation, point mutations, downregulation or loss of outer membrane porins, upregulation of drug efflux pumps, and acquisition of β-lactamases [6,8,9]. In 2018, the World Health Organization released a priority list of pathogens to target for the discovery of novel antimicrobial solutions, and carbapenem-resistant *P. aeruginosa* was listed as a critical priority in the top tier [10]. Treating an infection caused by MDR *P. aeruginosa* comes at a high cost, with both a significantly higher risk of morbidity and a treatment cost estimated between $25,300–$32,680 more for resistant infections compared to susceptible infections [7]. With a scarcity of antibiotics to combat MDR *P. aeruginosa*, alternative therapeutics are being investigated on their own and as antibiotic adjuvants [11].

A potential alternative therapeutic for treating *P. aeruginosa* infections is lytic bacteriophages (phages). Phages are viruses that specifically bind to, infect, and lyse bacteria. First discovered by Frederick Twort in 1915 and Felix D’Herelle in 1917, the clinical potential of phages was quickly recognized, and phages were successfully used in the treatment of both animal and human bacterial infections in the early 1920s [12]. After the invention of antibiotics, phage therapy lost popularity in the West, but with the rising rates of antibiotic resistance, there has been a renewed interest in the field [13]. Phage therapy is extremely specific, allowing for the selection of phages that kill only pathogenic bacteria and do not target normal microflora. Several studies both in vitro and in vivo have shown the efficacy of various phages in killing *P. aeruginosa* [14]. Hall et al., investigated the use of a phage cocktail against *P. aeruginosa* and found a significant reduction in bacterial densities in vitro and in wax moth larvae [15]. Phage treatment of *P. aeruginosa*-infected mice showed increased survival and decreased bacterial burden [16,17,18,19].

While phage therapy on its own can target and kill bacteria, regulatory hurdles and safety questions have slowed its progress towards clinical trials [20]. As antibiotics are the current standard of care, using phages as an adjuvant to antibiotics instead of on their own may be a more relevant way to apply phage therapy in treating MDR infections [21]. In vitro and mouse model studies have elucidated several phage antibiotic combinations, capable of reducing the bacterial burden more than the additive effects of either treatment alone, termed phage–antibiotic synergy [22,23]. Additionally, the use of phages in combination with antibiotics has been shown to re-sensitize resistant bacteria to antibiotics [24,25].

While not yet approved by the U.S. Food and Drug Administration (FDA), phages in combination with antibiotics have been used in various emergency clinical cases to treat infections caused by MDR *P. aeruginosa*. Using a phage that binds to a protein of a drug-efflux pump in combination with ceftazidime showed efficacy against *P. aeruginosa* infection both in mice and in a human case of endocarditis [25,26]. Additionally, a recurrent urinary tract infection treated with a phage cocktail in combination with meropenem and colistin resulted in complete infection clearance with no bacteria detected at one year post-treatment [27]. With treatment options becoming increasingly limited due to antibiotic resistance, phage antibiotic combinations need to be further explored for their potential to curb the threat posed by MDR *P. aeruginosa* infections. The purpose of this study was to investigate the potential synergy of a 5-phage cocktail, PAM2H, in combination with antibiotics (ceftazidime, ciprofloxacin, gentamicin, meropenem) against MDR clinical isolates of *P. aeruginosa*.

## 2. Results & Discussion

### 2.1. Phage Cocktail PAM2H Re-Sensitizes MDR P. aeruginosa Strains to Antibiotics

In order to determine the level of susceptibility to antibiotics, phages or combination treatment, minimum inhibitory concentrations (MICs) and fractional inhibitory concentrations (FICs) were measured for PAO1, PAO1::*lux* and 14 phylogenetically diverse MDR *P. aeruginosa* clinical strains. The MIC was determined to be the lowest concentration of antibiotic or phage where there was no visible bacterial growth (Table 1). All of the *P. aeruginosa* diversity set strains and PAO1::*lux* were classified as resistant to at least two of the tested antibiotics according to Clinical and Laboratory Standards Institute (CLSI) resistance breakpoints (Table 2) [28]. The difference in resistant profiles between PAO1 and PAO1::*lux* is due to the inclusion of a gentamicin marker on the lux cassette inserted in the PAO1::*lux* chromosome. The initial experiments were completed using PAO1 because this strain is widely characterized, allowing for comparison of our results to a vast array of previously published data. However, follow-on studies were completed using genetically diverse MDR clinical strains to assess whether our PAM2H phage cocktail has synergistic activity with antibiotics to which the bacterial target strain is resistant.

FIC assays were used to determine the susceptibility of the MDR strains to antibiotics in the presence of PAM2H. Each of the 16 *P. aeruginosa* strains was tested with decreasing concentrations of the PAM2H phage cocktail and antibiotic to determine the lowest concentration of antibiotics that could inhibit growth in the presence of PAM2H (Table 3). The concentration of CAZ in the presence of PAM2H that completely inhibited growth (MIC_ABΦ_) was 256-fold lower than the MIC of CAZ alone for 12 of 16 strains. Similarly, for MEM, GEN and CIP, the MIC_ABΦ_ was at least 64-fold lower than the MIC for 9, 12, and 12 strains, respectively. Importantly, the presence of PAM2H not only reduced the antibiotic MIC but also rendered the strains susceptible to these antibiotics based on CLSI guidelines (Table 2). In the presence of the PAM2H cocktail, the number of strains susceptible to CAZ increased by 63% and the number of strains susceptible to MEM, GEN, and CIP increased by 56%, 31%, and 81%, respectively. These data suggest that including PAM2H in combination treatment with an antibiotic that the bacterial target is resistant to could result in efficacious outcomes by re-sensitizing the bacterial strain to the antibiotic.

The FIC for each antibiotic, FIC_AB_, was calculated for the lowest concentration of antibiotic that could completely inhibit growth in the presence of the PAM2H cocktail using the equation: FIC_AB_ = MIC_ABΦ_/MIC_AB_ (Table 4) [29]. As the standard error in the microdilution MIC assay is one dilution on either side of the MIC value, an MIC_ABΦ_ value greater than two-fold below the MIC_AB_ would be considered a significant change in bacterial strain susceptibility to the antibiotic. FIC_AB_ was chosen over applying a standard FIC index because phages replicate in bacteria; thus, the phage concentration changes over time. Fifteen of 16 strains had a significant increase in susceptibility to at least one antibiotic in the presence of PAM2H, and 12 of those strains had increased susceptibility to all four antibiotics. This indicates that in vitro, PAM2H has the ability to significantly increase the sensitivity of MDR *P. aeruginosa* strains to multiple different antibiotics. This increase in susceptibility across multiple classes of antibiotics in the presence of phages is important clinically, as it would give more options for treatment regimens and would limit the use of “drugs of last resort” [25,30].

### 2.2. PAM2H + CAZ Combination Treatment Enhances Efficacy in a Mouse Dorsal Wound Model

**Figure 1:**
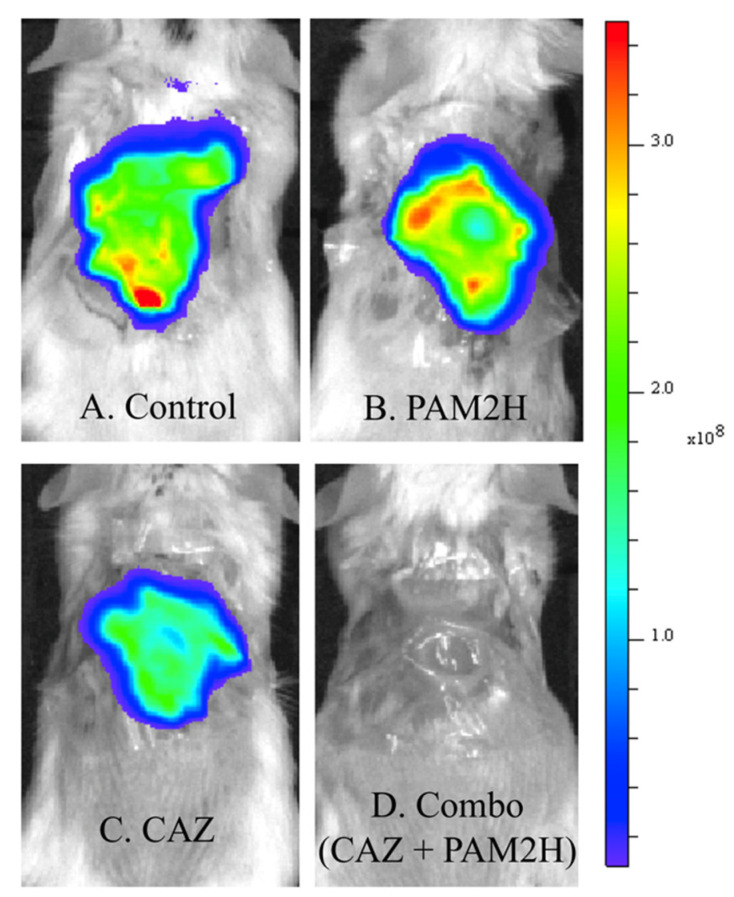
IVIS images showing the radiance (p/s/cm^2^/sr) of PAO1::*lux* bacteria in dorsal wounds of mice on day 4 post-surgery after completion of treatments. (**A**). IVIS of a mouse from the control-treated group receiving only PBS, (**B**). PAM2H (phage cocktail) only treated group, (**C**). CAZ only treated group, (**D**). combination treated group receiving both PAM2H and CAZ.

**Figure 2:**
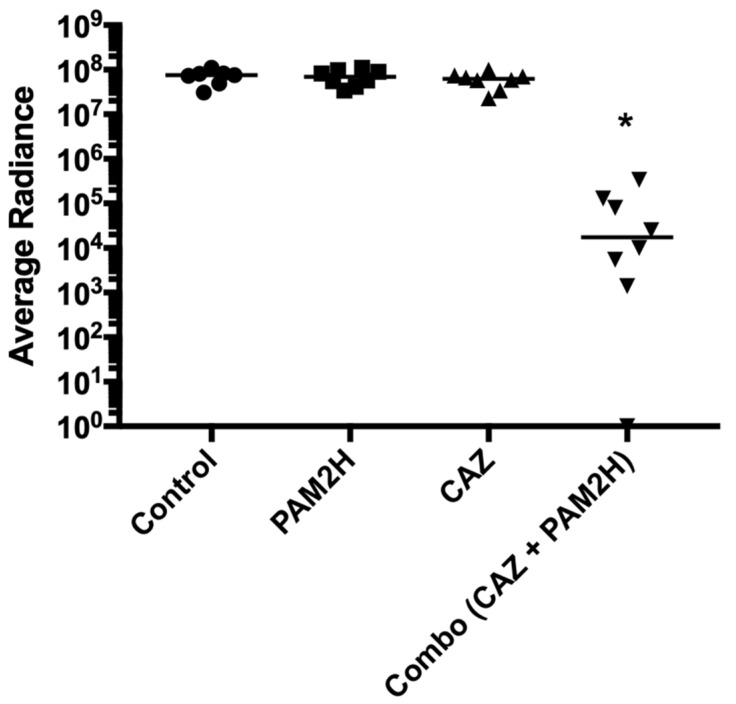
Quantification of the bacterial luminescence for mice from each treatment group as determined by IVIS on day 4 at the completion of treatment. * *p* < 0.05.

The in vitro results described above revealed that the CAZ MIC for PAO1 was reduced 256-fold in the presence of PAM2H. We hypothesized that this significant increase in in-vitro efficacy would result in increased efficacy in vivo in a mouse dorsal wound model compared to antibiotic treatment alone. To test this hypothesis, mice were wounded dorsally and infected with PAO1::*lux*. Mice were then treated with either PBS (control), PAM2H phage cocktail, CAZ, or PAM2H and CAZ in combination, as described in Section 3. The CAZ treatment concentration was determined by calculating the mouse equivalent dose based on the 2 g/dose of CAZ recommended for treating complex infections [31]. On day 4, 24 h after treatment was ceased, the radiance (p/s/cm^2^/sr) of the bioluminescent signal of PAO1::*lux* in the combo-treated mice appeared significantly reduced compared to the other groups, as shown in the heat maps in Figure 1. When quantified, the bioluminescence was significantly reduced compared to the other treatment groups (Figure 2), indicating a reduction in bacterial counts in the wounds of combo-treated mice.

**Figure 3:**
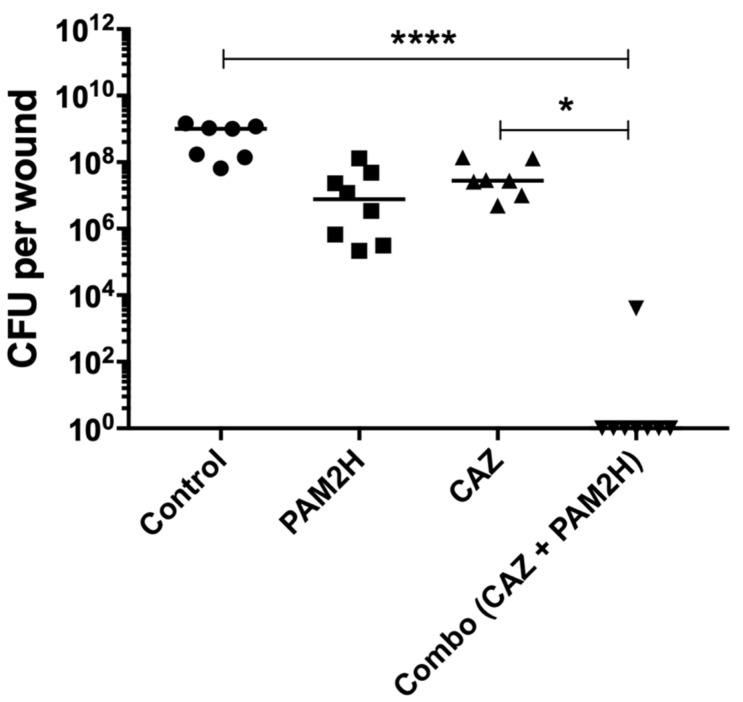
Colony forming units obtained from excised wound tissue on day 4 post treatment. * *p* < 0.05, **** *p* < 0.0001.

To confirm these results, mice were euthanized on day 4, and wound tissues were collected and plated for colony forming units (CFU) (Figure 3). The PBS control mice had ~10^9^ CFU per wound at the time of collection. Phage-only and CAZ-only treated mice had a 1–2 log reduction, with CFU per wound at approximately 10^7^. The combo-treated wounds had a marked reduction in CFU compared to all groups, with no detectable CFU in seven out of the eight wounds collected, and a 5-log reduction in CFU compared to the PBS control for the remaining mouse wounds. These data indicate that the combination treatment resulted in a synergistic reduction in the bacterial burden compared to either treatment alone, as the median log reduction was greater than the sum of the median reductions of each monotherapy [23]. Additionally, all of the treatments had increased survival as compared to the PBS control-treated group. In CFU count experiments, only 47% of the PBS-treated mice survived to day 4, whereas all of the mice in the treatment groups survived.

**Figure 4:**
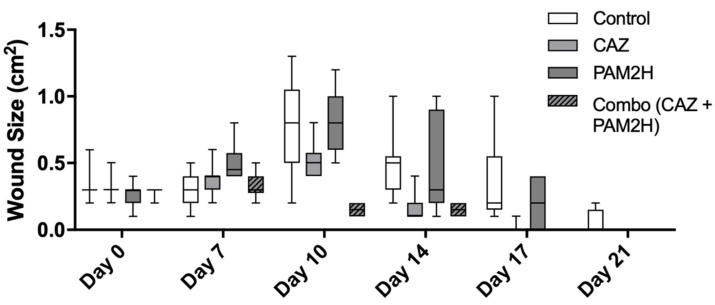
Mouse dorsal wound sizes as measured by an Aranz wound measurement device over 21 days. On day 10, the wound size for the combo-treated group was significantly smaller than that in the control or in the other treatment groups (*p* < 0.005), and the median wound size on day 10 for the combo group was less than the median wound size on day 0, meaning the wounds had begun to contract and heal.

Finally, as a further assessment of the efficacy of this model, physical wound size was measured over the course of the 21-day experiment (Figure 4). Typically in this model, after the Tegaderm bandage is removed (day 7 for this study), the wound significantly increases in size, peaking at day 10 before contraction and healing begin [32]. The wounds of both CAZ- and combo-treated mice closed on day 21 of the experiment. On day 10 of this study, the wound size for the combo-treated group was significantly smaller than that in the control or in the other treatment groups (*p* < 0.005). Additionally, the median wound size on day 10 for this group was less than the median wound size on day 0, meaning the wounds did not increase in size following Tegaderm removal, but began to contract and heal. This suggests that the combo treatment prevented the spread of bacteria into and necrosis of the surrounding tissue of the wound. While the final wound closure rate for the combination treatment was the same as that for the CAZ treatment, the reduced necrosis and lack of wound expansion indicate that combination treatment can help to prevent the spread of bacteria to other tissues and can reduce the amount of future scar tissue, leading to better clinical outcomes.

### 2.3. Recovered Strain Virulence in Wax Worms

**Figure 5:**
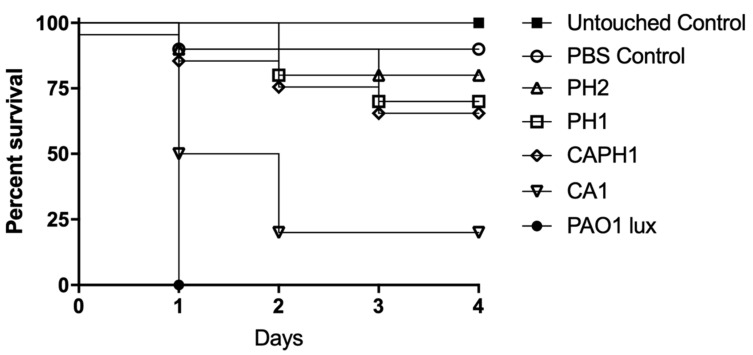
Kaplan–Meier survival curve for *Galleria* wax worms inoculated with PAO1::*lux* mutants recovered from mouse dorsal wounds post-treatment. Original PAO1::*lux* was used as a positive control, and an untouched group and PBS injected group served as negative controls. PH1 and PH2 were isolates obtained from mice in the phage-only treatment group. CA1 was collected from the CAZ-only treatment group, and CAPH1 was obtained from the combination treatment group. PH2, PH1, and CAPH1 showed a significant increase in survival compared to the PAO1::*lux* (*p* < 0.0001). Note: the survival curves for PH1 and CAPH1 were identical; thus, the survival curve for CAPH1 was moved below PH1 so that the symbols for each curve are discernible on the graph.

Several of the bacterial colonies recovered on day 4 from the above mouse wound experiments showed morphological differences from the parental strain PAO1::*lux*. Four isolates, PH1 to PH4, were selected from two mice in the PAM2H-only treatment group, four isolates, CA1 to CA4, were selected from two mice in the CAZ-only treatment groups, and two isolates, CAPH1 and CAPH2, were recovered from one mouse in the combo treatment group. The isolates obtained from the one combo-treated mouse wound had different morphologies. No colonies from the control group showed morphological differences from the parental strain. These PAO1::*lux* mutant isolates were assessed for virulence in the *G. mellonella* larva model. Decreased virulence was observed in four of the 10 mutants as compared to the original PAO1::*lux* control (Figure 5). All of the wax worms infected with wild type PAO1::*lux* died within 24 h of inoculation, while worms inoculated with mutant strains PH1, PH2, CA1, and CAPH1 demonstrated increased survival. Of the mutant strains, PH2 showed the greatest decrease in virulence as compared to PAO1::*lux*, with 80% survival 4 days post infection (*p* < 0.0001); PH1 and CAPH1 showed a 70% survival (*p* < 0.0001). The CA1 mutant strain showed a 20% survival 4 days post infection; this increase in survival was not statistically significant. The decrease in virulence of some of the recovered strains suggests that while there were bacteria remaining in the mouse wounds after treatment, their ability to cause a robust infection was reduced. This was confirmed by the increased survival of mice in the treatment groups compared to the control group. This decrease in virulence was further investigated by sequencing the mutant strains to determine which virulence factors may be impacted by treatment with phage, antibiotics, or combination treatment.

### 2.4. Whole-Genome Sequencing of Recovered PAO1 Strains

Genomic DNA from PAO1::*lux* variants recovered from the mouse dorsal wound experiments post treatment was sequenced to compare to the original PAO1::*lux*. Every strain from the phage-only treatment had mutations to at least one gene coding for a known phage receptor. However, the bacteria recovered from the mouse given combination phage–antibiotic treatment had no SNP changes in typical phage receptor genes (Table 5). This suggests that treatment with phages and antibiotics concurrently is a better option than treatment with phages alone, because it may reduce the emergence of phage resistance. Additionally, PH2 and CAPH1 were observed to be missing the mexX gene due to nonsynonymous large deletion events. The mexX gene is an antimicrobial resistance (AMR) gene encoding a periplasmic protein that is part of a drug efflux pump. None of the strains from the antibiotic-only treatment had this mutation. One or more of the phages in the PAM2H cocktail may select against drug efflux pumps, helping to re-sensitize *P. aeruginosa* to antibiotic treatment. This mutation was correlated with a significant decrease in strain virulence as seen in Figure 5, indicating that a functional MexX protein may be important for virulence in PAO1 [33].

## 3. Materials and Methods

### 3.1. Bacterial Strains and Bacteriophages

*P. aeruginosa* strain PAO1, a wound isolate, was selected because of its extensive characterization and wide use as a laboratory strain [34]. PAO1::*lux* was created by cloning the *luxCDABE* luciferase-production operon into the chromosome of PAO1 using a Tn*7* plasmid, as previously described [35]. For in vitro testing, 14 additional MDR *P. aeruginosa* clinical strains susceptible to the PAM2H bacteriophage cocktail were selected from a 100-strain panel of genetically diverse isolates generously provided by the Multidrug-resistant organism Repository and Surveillance Network (MRSN, WRAIR). The *P. aeruginosa* phage cocktail PAM2H contains equal concentrations of five unique phages: EPa5, EPa11, EPa15, EPa22, and EPa43 [36] (Table 6).

### 3.2. Bacteriophage Isolation and Cocktail Preparation

Bacteriophages were propagated on *P. aeruginosa* strains PAO1 or MRSN1680. The host bacteria were grown in heart infusion broth (HIB; Becton, Dickinson and Co., Franklin Lakes, NJ) supplemented with 5 mM calcium chloride and incubated in a vented culture flask at 37 °C and 200 rpm. Phage stock lysate was added to 250 mL of an early exponential phase bacterial culture grown in HIB (OD600 of 0.1–0.2; 10^8^ colony forming units/mL) at a multiplicity of infection of 0.1 (10^7^ plaque forming units/mL) and incubated in a 500-mL plastic Erlenmeyer flask at 37 °C and 200 rpm overnight. The phage and bacterial debris were pelleted by centrifugation at 5500× *g* overnight, and then the phage was purified with 1-octanol (Sigma, St. Louis, MO, USA) washes and a cesium chloride (Sigma) density gradient. The final phage concentrate was exchanged into gelatin-free SM buffer (100 mM sodium chloride, 50 mM Tris-HCl, pH 7.5, and 10 mM magnesium sulfate) by serial washes in a protein concentrator (Thermo Fisher, Waltham, MA, USA). Endotoxin levels were tested with the Endosafe-PTS device (Charles River Laboratories, Wilmington, MA, USA), and if needed, further purified using EndoTrap bulk resin (Hyglos GmbH, Bernried am Starnberger See, Germany) according to the manufacturer’s protocol, to ensure that the endotoxin level was below 500 EU per 10^9^ PFU (plaque-forming units).

### 3.3. Antibiotics

Antibiotics were selected from three classes based on their mechanism of action and clinical relevance: ceftazidime, generic name Tazicef (CAZ; Hospira, Lake Forest, IL, USA), ciprofloxacin (CIP; Sigma), gentamicin (GEN; Sigma), and meropenem (MEM; Sigma). Antibiotics were dissolved at time of use in a 10 mg/mL stock in 1× PBS (Thermo Fisher) except for ciprofloxacin which was dissolved in 0.1 N HCl (Sigma). Stocks were further diluted for treatments in 1× PBS.

### 3.4. Minimum Inhibitory Concentration Assays

Assays to determine the minimum inhibitory concentration (MIC) for antibiotics and bacteriophages were performed in accordance with the Clinical and Laboratory Standards Institute (CLSI) broth microdilution protocol [37] with the following modifications for the phage assay [38]. Briefly, to make the bacterial inoculum, individual colonies from test strains grown overnight on 1.5% HIB agar plates were suspended in deionized (DI) water (Thermo Fisher) to 0.5 McFarland standard, and 10 µL of the inoculated water was transferred to 11 mL of cation-adjusted Mueller Hinton Broth (CAMHB) (Thermo Fisher). To prepare the phage treatment, the PAM2H phage cocktail was diluted serially, 1:10 from 2 × 10^9^ PFU/mL to 2 PFU/mL in CAMHB. Fifty microliters of bacterial inoculum was added to wells of a 96-well microtiter plate, and 50 µL of phage dilutions was added to appropriate wells. One row of bacterial inoculum received no phages to serve as a positive growth control, and one row of only CAMHB served as a negative growth control. Plates were incubated at 37 °C overnight. The MIC for each phage or antibiotic was determined as the lowest concentration of treatment with no visible bacterial growth.

### 3.5. Fractional Inhibitory Concentration Assays

The fractional inhibitory concentrations (FIC) of antibiotics (CAZ, CIP, GEN, and MEM) in the presence of PAM2H were determined using the checkerboard method [39,40]. Briefly, antibiotics were serially diluted 1:2 from 1024 µg/mL down to 0.007813 µg/mL in CAMHB. PAM2H was serially diluted 1:10 starting at 4 × 10^9^ PFU/mL in CAMHB. To make the bacterial inoculum, individual colonies from test strains grown overnight on HIB agar plates were suspended in DI water to 0.5 McFarland standard, and 10 µL of the inoculated water was transferred to 11 mL of CAMHB. For each strain, 25 µL of antibiotic in decreasing concentrations from left to right was added to columns 1 to 10 starting at 8× MIC. Twenty-five microliters of PAM2H was added to each row of columns 1–10, decreasing from top to bottom starting at 40× MIC. Fifty microliters of bacterial inoculate was added to each well in columns 1–11, and 100 µL of only CAMHB medium was aliquoted to each well in column 12 as a negative growth control. Plates were incubated at 37 °C overnight. The MIC for the antibiotic in the presence of the phage was determined as the lowest concentration of antibiotic with no visible bacterial growth. Changes in antibiotic MIC in the presence of the phage greater than twofold (standard error of the assay) compared to the antibiotic MIC were considered significant.

### 3.6. Assessment of Phage Antibiotic Combination Treatment in a P. aeruginosa Mouse Wound Model

The PAM2H + CAZ combination treatment was assessed in our previously described mouse wound model [32,41]. Six-week-old female BALB/c mice were anesthetized with 130 mg/kg ketamine and 10 mg/kg xylazine, their backs were shaved, and a 6-mm, full-thickness wound was created on their dorsal side. Each wound was inoculated with approximately 1 × 10^7^ CFU (colony-forming units) of PAO1::*lux* and covered with a Tegaderm^TM^ bandage (3M, St. Paul, MN, USA). Mice were single-housed from day 0 (inoculation) through day 9. There were four groups of mice in the experiment: PBS control, phage treatment only, CAZ treatment only, and phage + CAZ (combo) treatment (Table 7). The phage and CAZ were both suspended in PBS to the required titer/concentration. Treatments were given as follows (Table 7): For PBS control, mice received 25 µL of PBS topically under the Tegaderm^TM^ dressing, on top of the wound, once a day, and a dose of PBS intraperitoneally (IP) at 5 µL/g body weight twice a day. For phage treatment only, mice received 25 µL of 1 × 10^8^ PFU phage topically under the Tegaderm^TM^ dressing, on top of the wound, once a day, and a dose of PBS IP at 5 µL/g body weight twice a day. For CAZ treatment only, mice received 25 µL of PBS topically under the Tegaderm^TM^ dressing, on top of the wound, once a day, and a 410 mg/kg dose of CAZ IP at 5 µL/g body weight twice a day. For combo treatment, mice received 25 µL of 1 × 10^8^ PFU phage topically under the Tegaderm^TM^ dressing, on top of the wound, once a day, and a 410 mg/kg dose of CAZ IP at 5 µL/g body weight twice a day.

Treatments were administered starting at 4 h post-infection. Phage treatments were given at 4 h (day 0), and then once daily on days 1–3, for a total of four doses of the phage. CAZ treatments were given at 4 h (day 0), and then twice daily, every 12 h, on days 1–3, for a total of seven doses of CAZ. On day 7 post-infection, Tegaderm^TM^ dressings were removed, and the wounds were left exposed to air for the remainder of the experiment.

Multiple outcomes were measured to assess the above treatments, including weight and clinical score, wound size and healing, and the bioluminescent signal and CFU of the bacterial burden in the wound. Mouse weights and clinical scores were monitored and recorded daily through day 6 of the experiment. An in vivo imaging system (IVIS; PerkinElmer, Waltham, MA, USA) was used to measure the bioluminescent signal of PAO1::*lux* as a means to visualize and perform relative quantification of the bacterial burden in the wound beds over the course of the experiment (control mice *n* = 7, treatment groups *n* = 8). To do so, mice were anesthetized with isoflurane gas and placed dorsal side up, inside the IVIS chamber. Bioluminescence measurements were taken with an exposure time of 1 min on days 1 and 4. Using Living Image Software version 4.2 (PerkinElmer), pictures were analyzed and bioluminescence was quantified. To determine the average radiance, each wound was measured using a region of interest (ROI) of 2.3 cm^2^. To confirm the IVIS data, the bacteria in the wound beds were quantified on day 4 post-infection by excising the wounds and plating for CFU (control mice *n* = 7, treatment groups *n* = 8). To do this, the mice were humanely euthanized using CO_2_ exposure followed by cervical dislocation, and the wound and surrounding tissue (~3 mm) were excised and placed in PBS. The tissue was homogenized, 10-fold serially diluted, and plated on HIB agar. CFU were enumerated following overnight incubation at 37 °C. In additional studies, the wound size was measured and compared for all mice in all treatment groups using a Silhouette wound measurement device (Aranz Medical Ltd., Christchurch, New Zealand) on days 0, 10, 14, 17, and 21 post-infection (control *n* = 9, PAM2H *n* = 10, CAZ *n* = 20, combo *n* = 10). Statistical analyses were completed using a Kruskal–Wallis test followed by Dunn’s multiple comparison test. Significance was established at *p* < 0.05.

Research was conducted in an AAALACi accredited program in compliance with the Animal Welfare Act and other federal statutes and regulations relating to animals and experiments involving animals and adhered to principles stated in the Guide for the Care and Use of Laboratory Animals [42]. The study protocol was reviewed and approved by the Walter Reed Army Institute of Research/Naval Medical Research Center Institutional Animal Care and Use Committee in compliance with all applicable Federal regulations governing the protection of animals in research.

### 3.7. Assessment of P. aeruginosa Strain Virulence in Galleria mellonella Larvae

Colonies of *P. aeruginosa* PAO1::*lux* isolated from mouse wounds after treatment with PAM2H phages only (*n* = 4), CAZ only (*n* = 4), or the PAM2H + CAZ combination (*n* = 2) displayed different phenotypes and colony morphologies compared to the parental strain. To determine if any of these colonies were attenuated, we assessed bacterial virulence in a *G. mellonella* larva (wax worm) model of infection, as previously described [43]. *P. aeruginosa* strains were grown to the exponential phase, washed, and resuspended in PBS to approximately 1 × 10^7^ CFU per mL. Wax worms (Vanderhorst, Inc., St. Marys, OH, USA) in the final-instar larval stage and weighing 200–300 mg were saved and housed in clean plastic Petri dishes, 10 worms per group. Worms in each group were inoculated with 5 µL of one bacterial strain into their last left proleg using a 10-µL Hamilton syringe (Fisher Scientific, Pittsburgh, PA, USA). After infection, worms were incubated in plastic Petri dishes at 37 °C and monitored for death over four days. Worms were considered dead when they displayed no movement in response to tactile stimuli. Two control groups were included in the experiment, an “untouched” control group that did not receive any injections, to ensure the health of the worms after shipping, and a PBS control group that was injected with PBS instead of bacteria, to control for detrimental effects from injection. Survival curves were compared using the Mantel–Cox test with Bonferroni’s correction for multiple comparisons. Significance was established at *p* < 0.05.

### 3.8. P. aeruginosa DNA Isolation and Whole-Genome Sequencing

DNA was extracted using the DNeasy UltraClean Microbial Kit (Qiagen, Germantown, MD, USA), and libraries were constructed using the KAPA HyperPlus Library preparation kit (Roche Diagnostics, Indianapolis, IN, USA). Libraries were quantified using the KAPA Library Quantification Kit—Illumina/Bio-Rad iCycler™ (Roche Diagnostics). Sequencing was performed using an Illumina MiSeq desktop sequencer (Illumina Inc., San Diego, CA, USA) with a MiSeq Reagent Kit v3 (600 cycle) (Illumina Inc.).

Species identification and contamination detection were performed from sequencing reads using Kraken2 [44]. Reads were trimmed for adapter content and quality with BBduk [45] followed by *de novo* assembly using Newbler v2.9 [46]. Antimicrobial resistance genes were annotated using a combination of ARIBA [47] and AMRFinderPlus [48]. MLST assignment was performed using multilocus sequence typing [49] against the relevant schema hosted by pubMLST [50]. The progenitor PAO1::*lux* draft assembly was error-corrected and annotated using a combination of Snippy [51], Pilon [52], and Prokka [53] for use as the SNP analysis reference genome. Snippy was used to identify single nucleotide polymorphisms (SNPs) in each of the passaged isolates with respect to the annotated reference. Further comparative genomic analyses were performed using Geneious Prime (Biomatters, Auckland, New Zealand).

## 4. Conclusions

Increasing threats from MDR pathogens have made it necessary to explore alternative treatment options. Phage–antibiotic combination therapy is a promising candidate for combating MDR *P. aeruginosa* infections. The studies described here show that using *P. aeruginosa* phages in combination with different classes of antibiotics was not only efficacious, but synergistic in the reduction of bacterial populations and resulted in the re-sensitization of MDR *P. aeruginosa* to antibiotics. Bacteria remaining in mouse wounds following combination treatment were shown to have mutations in virulence-associated drug efflux pump genes, suggesting that the only way for the bacteria to evade the combination treatment was to become avirulent, which would allow the host immune system to clear the infection. Additionally, while bacteria remaining in the phage-only treated mouse wounds had mutations for phage receptors, and were thus resistant to phage infection, none of these mutations was found in the combination treatment bacteria, suggesting that the combination treatment reduced the incidence of the bacteria becoming resistant to the phage treatment. Taken together, these data show the synergy that exists with phage–antibiotic combination treatment and support the idea that these treatment regimens would enhance the efficacy of both phages and antibiotics in a human patient and would result in better clinical outcomes.

## Figures and Tables

**Table 1:** Minimum inhibitory concentration results for antibiotics and phages in different *P. aeruginosa* strains.

Isolate No.	Source	MIC (µg/mL)				MIC (PFU/mL)
		CAZ	MEM	GEN	CIP	PAM2H
MRSN321	Wound	32	16	2	0.025	1 × 10^0^
MRSN994	Respiratory	32	32	4	4	1 × 10^6^
MRSN2108	Tissue	16	64	2	8	>1 × 10^9^
MRSN5498	Tissue	16	32	256	8	1 × 10^8^
MRSN5508	Fluid	16	32	128	8	1 × 10^8^
MRSN5519	Wound	32	32	128	32	1 × 10^6^
MRSN6695	Urine	128	32	8	2	>1 × 10^9^
MRSN8915	Urine	4	32	16	64	1 × 10^4^
MRSN11538	Wound	8	32	128	4	1 × 10^9^
MRSN12282	Respiratory	32	64	128	64	1 × 10^2^
MRSN15678	Wound	16	16	128	32	1 × 10^6^
MRSN16345	Urine	16	8	1	32	1 × 10^0^
MRSN23861	Respiratory	16	256	2	32	1 × 10^7^
MRSN409937	Fluid	128	16	2	8	1 × 10^0^
PAO1::*lux* *	Laboratory	2	1	32	1	1 × 10^0^
PAO1	Laboratory	1	2	1	1	1 × 10^0^

* PAO1::*lux* has a different resistance profile from PAO1 due to the inclusion of a gentamicin resistance marker on the inserted lux (luciferase) gene cassette.

**Table 2:** CLSI MIC breakpoints for classifying antibiotic resistance in *P. aeruginosa* strains.

Antibiotic	CLSI MIC Breakpoints µg/mL		
	Susceptible	Intermediate	Resistant
Ceftazidime (CAZ)	≤8	16	≥32
Ciprofloxacin (CIP)	≤0.5	1	≥2
Gentamicin (GEN)	≤4	8	≥16
Meropenem (MEM)	≤2	4	≥8

**Table 3:** Fractional inhibitory concentrations of antibiotics CAZ, MEM, GEN, and CIP in the presence of the PAM2H phage cocktail.

Isolate No.	Ceftazidime (CAZ)			Meropenem (MEM)			Gentamicin (GEN)			Ciprofloxacin (CIP)		
	MIC (µg/mL)	MIC in the Presence of PAM2H (µg/mL)	Amount of PAM2H (PFU/mL)	MIC (µg/mL)	MIC in the Presence of PAM2H (µg/mL)	Amount of PAM2H (PFU/mL)	MIC (µg/mL)	MIC in the Presence of PAM2H (µg/mL)	Amount of PAM2H (PFU/mL)	MIC (µg/mL)	MIC in Presence of PAM2H (µg/mL)	Amount of PAM2H (PFU/mL)
PA321	32	0.0625	1 × 10^0^	16	0.0625	1 × 10^1^	2	0.007813	1 × 10^1^	0.25	0.000976563	1 × 10^0^
PA994	32	0.125	1 × 10^7^	32	0.125	1 × 10^7^	4	0.015625	1 × 10^7^	4	0.015625	1 × 10^7^
PA2108	16	0.0625	1 × 10^4^	64	0.25	1 × 10^3^	2	0.007813	1 × 10^3^	8	0.03125	1 × 10^3^
PA5498	16	2	1 × 10^5^	32	0.25	1 × 10^7^	256	0.5	1 × 10^7^	8	0.03125	1 × 10^7^
PA5508	16	0.0625	1 × 10^5^	32	0.125	1 × 10^2^	128	0.5	1 × 10^2^	8	0.03125	1 × 10^6^
PA5519	32	0.125	1 × 10^5^	32	0.125	1 × 10^6^	128	0.5	1 × 10^6^	32	0.125	1 × 10^5^
PA6695	128	128	1 × 10^2^	32	32	1 × 10^2^	8	8	1 × 10^2^	2	1	1 × 10^2^
PA8915	4	0.015625	1 × 10^0^	32	16	1 × 10^0^	16	16	1 × 10^0^	64	0.25	1 × 10^0^
PA11538	8	8	1 × 10^7^	32	16	1 × 10^2^	128	16	1 × 10^3^	4	0.25	1 × 10^7^
PA12282	32	0.125	1 × 10^1^	64	16	1 × 10^0^	128	2	1 × 10^0^	64	0.25	1 × 10^0^
PA15678	16	0.0625	1 × 10^5^	16	2	1 × 10^4^	128	8	1 × 10^5^	32	0.125	1 × 10^5^
PA16345	16	0.0625	1 × 10^0^	8	0.125	1 × 10^1^	1	0.003906	1 × 10^0^	32	0.125	1 × 10^0^
PA23861	16	16	1 × 10^3^	256	256	1 × 10^2^	2	0.007813	1 × 10^7^	32	32	1 × 10^8^
PA409937	128	0.5	1 × 10^0^	16	2	1 × 10^1^	2	0.015625	1 × 10^1^	8	0.03125	1 × 10^0^
PAO1::*lux*	2	0.007813	1 × 10^0^	1	0.003906	1 × 10^0^	32	0.125	1 × 10^0^	1	0.003906	1 × 10^0^
PAO1	1	0.003906	1 × 10^0^	2	0.03125	1 × 10^0^	1	0.003906	1 × 10^0^	1	0.003906	1 × 10^0^

MIC in the presence of PAM2H is the lowest concentration of antibiotic with PAM2H where there was no visible bacterial growth as determined by checkerboard assay. Amount of PAM2H column is the PFU/mL of the corresponding antibiotic well. Cells are color-coded by the MIC value: red is resistant, yellow is intermediate, green is susceptible.

**Table 4:** FIC_AB_ values for four antibiotics against 16 *P. aeruginosa* strains.

	FIC_AB_			
Isolate No.	CAZ	MEM	GEN	CIP
MRSN321	0.002	0.004	0.004	0.039
MRSN994	0.001	0.004	0.004	0.004
MRSN2108	0.004	0.004	0.004	0.004
MRSN5498	0.125	0.008	0.002	0.004
MRSN5508	0.004	0.004	0.004	0.004
MRSN5519	0.004	0.004	0.004	0.004
MRSN6695	1	1	1	0.5
MRSN8915	0.004	0.5	1	0
MRSN11538	1	0.5	0.125	0.063
MRSN12282	0.004	0.25	0.016	0.004
MRSN15678	0.004	0.125	0.063	0.004
MRSN16345	0.004	0.016	0.004	0.004
MRSN23861	1	1	0.004	1
MRSN409937	0.004	0.125	0.008	0.004
PAO1::*lux*	0.004	0.004	0.004	0.004
PAO1	0.004	0.016	0.004	0.004

FIC_AB_ is an assessment of the change in a bacterial strain’s susceptibility to an antibiotic in the presence of a phage and is obtained by dividing the new MIC of the antibiotic (in the presence of PAM2H) by the MIC of the antibiotic by itself. A value of less than 0.5 indicates a significant increase in the bacterial strain’s susceptibility to the antibiotic and is highlighted in blue.

**Table 5:** Summary of observed small variant (SNPs and short indels) with respect to progenitor strain PAO1::*lux* observed in recovered mouse mutant strains and whether the mutation impacts a known phage receptor.

Product ^1^	Mutation ^2^	Impact ^3^	Known Phage Receptor ^4^	PAO1::*lux* Mutant Isolates									
				PH1	PH2	PH3	PH4	CA1	CA2	CA3	CA4	CAPH1	CAPH2
type 4 fimbrial biogenesis outer membrane protein PilQ	SNP (A > C)	Thr605Pro	Yes	+									
B-band O-antigen polymerase	Insertion (A)	Thr46 (fs)	Yes			+							
B-band O-antigen polymerase	SNP (A > G)	Tyr249Cys	Yes				+						
type 4 fimbrial biogenesis protein PilY1	Deletion (AGACCAGCTT)	Gln520 (fs)	Yes			+							
type 4 fimbrial biogenesis protein PilB	Deletion (A)	His414 (fs)	Yes				+						
type 4 fimbrial biogenesis protein PilB	Deletion (CGGA)	Arg258 (fs)	Yes		+								
glucose-6-phosphate isomerase	Insertion (A)	Thr219 (fs)	No	+									
oxidoreductase	SNP (T > A)	Ser133Thr	No	+	+	+	+	+	+	+	+	+	+

^1^ Gene product in which a mutation was observed. Gene products listed on multiple lines are indicative of distinct mutations being observed across isolates. ^2^ Description of the mutation event that was observed (Insertion, Deletion, or Single Nucleotide Polymorphism (SNP)). ^3^ Impact of the mutation on the translated protein product. (fs): a frameshift has occurred. ^4^ Whether or not the product impacted is a known bacteriophage receptor. + symbol marks the gene mutation that each strain contained.

**Table 6:** *P. aeruginosa* phages in the PAM2H cocktail.

Phage Name	Family	Genus
EPa5	Siphoviridae	*Abidjanvirus*
EPa11	Myoviridae	*Pbunavirus*
EPa15	Myoviridae	*Pbunavirus*
EPa22	Myoviridae	*Pbunavirus*
EPa43	Siphoviridae	*Abidjanvirus*

**Table 7:** Treatment type and frequency for mouse groups infected with PAO1::*lux.*

Treatment Groups	Treatment Location	
	Topical (25 µL)	Intraperitoneal (5 µL/g)
	1 × per day	2 × per day
Phage-Treated Group	PAM2H cocktail	PBS
Ceftazidime-Treated Group	PBS	Ceftazidime (CAZ)
Combination-Treated Group	PAM2H cocktail	Ceftazidime (CAZ)
Control-Treated Group	PBS	PBS

## Data Availability

Data sharing not applicable.
